# Emergence of a novel recombinant Seneca Valley virus in Central China, 2018

**DOI:** 10.1038/s41426-018-0183-1

**Published:** 2018-11-14

**Authors:** Zeng Wang, Xiaozhan Zhang, Ruoqian Yan, Panpan Yang, Yanyang Wu, Dongdong Yang, Chuanzhou Bian, Jun Zhao

**Affiliations:** 1 College of Animal Science and Veterinary Medicine, Henan Agricultural University, Zhengzhou, 450002 Henan China; 2 College of Veterinary Medicine, Henan University of Animal Husbandry and Economy, Zhengzhou, 450046 Henan China; 3 Henan Centre for Animal Diseases Control & Prevention, Zhengzhou, 450008 Henan China

To the Editor

Seneca Valley virus (SVV), an emerging non-enveloped, single-stranded RNA virus, belongs to the genus *Senecavirus* in the family *Picornaviridae* and has drawn a large amount of attention because of its pathogenicity to swine and oncolytic properties^1,2^. The genome of SVV is ∼ 7300 nucleotides (nt) in length, including a 5′-untranslated region (UTR), a large open reading frame (ORF) and a polyadenylated 3′-UTR. The ORF encodes a single large polyprotein, which is cleaved into 12 mature proteins in a typical picornavirus L-4-3-4 layout (5′-L-VP4-VP2-VP3-VP1-2A-2B-2C-3A-3B-3C-3D-3′)^3^. The structural proteins VP1, VP2, and VP3, lying on the outer capsid surface of the SVV virion, have been shown to co-interact with anthrax toxin receptor 1 to initiate the infection and further induce host cell antibody responses^4,5^. Only one serotype of SVV is recognized, but strain differences have been characterized. SVV strains have been classified into three temporal clades based on their virulence, genetic characteristics, and geographical distribution. Clade I contains SVV-001, and clade II contains SVV strains that circulated in the United States between 1988 and 1997 and are considered to have little or no pathogenicity to swine. Clade III includes SVV strains isolated from 2001 to 2018 that are associated with vesicular diseases^6^.

Recently, several SVV-associated vesicular diseases, characterized by lameness, coronary band hyperemia, vesicles on the snout and coronary bands, and increased neonatal mortality, have been reported in Canada and the United States^7,8^. SVV-associated vesicular disease is clinically indistinguishable from other swine vesicular diseases, such as foot-and-mouth disease (FMD), swine vesicular disease (SVD), and vesicular stomatitis (VS)^2,6^. SVV outbreaks have also been reported in several other countries, such as Brazil, China, Thailand, and Colombia, which suggests that the disease has become a worldwide problem^2,6^.

One hallmark of picornaviruses is the recombination, which helps a virus adapt to a new environment and evade the host antiviral immune system^9^. Previous studies have identified several variants generated by intraspecies recombination in the family *Picornaviridae*, including enteroviruses, aphthoviruses, and cardioviruses^9^. However, neither the extent of intraspecies recombination among SVVs, nor its role in the evolution and spread of the virus among pigs has been reported.

In January 2018, an outbreak of vesicular disease occurred at a pig farm in Henan, Central China. Eighteen out of 87 sows, which had been vaccinated with commercial foot-and-mouth disease virus (FMDV) vaccines, showed vesicle lesions on snouts and coronary bands. Meanwhile, 12 of the 18 sick sows presented with lameness and lethargy, but then recovered 2 weeks later. Vesicular lesion swab specimens were collected and analyzed by reverse transcription–polymerase chain reaction (RT-PCR) using specific primers for FMDV, swine vesicular disease virus (SVDV), vesicular stomatitis virus (VSV), vesicular exanthema of swine virus (VESV), porcine pegivirus (PPgV), and SVV. We successfully isolated an infectious SVV strain, HeN-1/2018, from the samples, which were negative for FMDV, SVDV, VSV, VESV, and PPgV, but positive for SVV. In this study, we characterized the genome of the newly identified SVV HeN-1/2018 strain. Genetic, evolutionary, and recombination analyses were performed to determine the genetic diversity of SVVs and the potential origin of the virus.

**Fig. 1:**
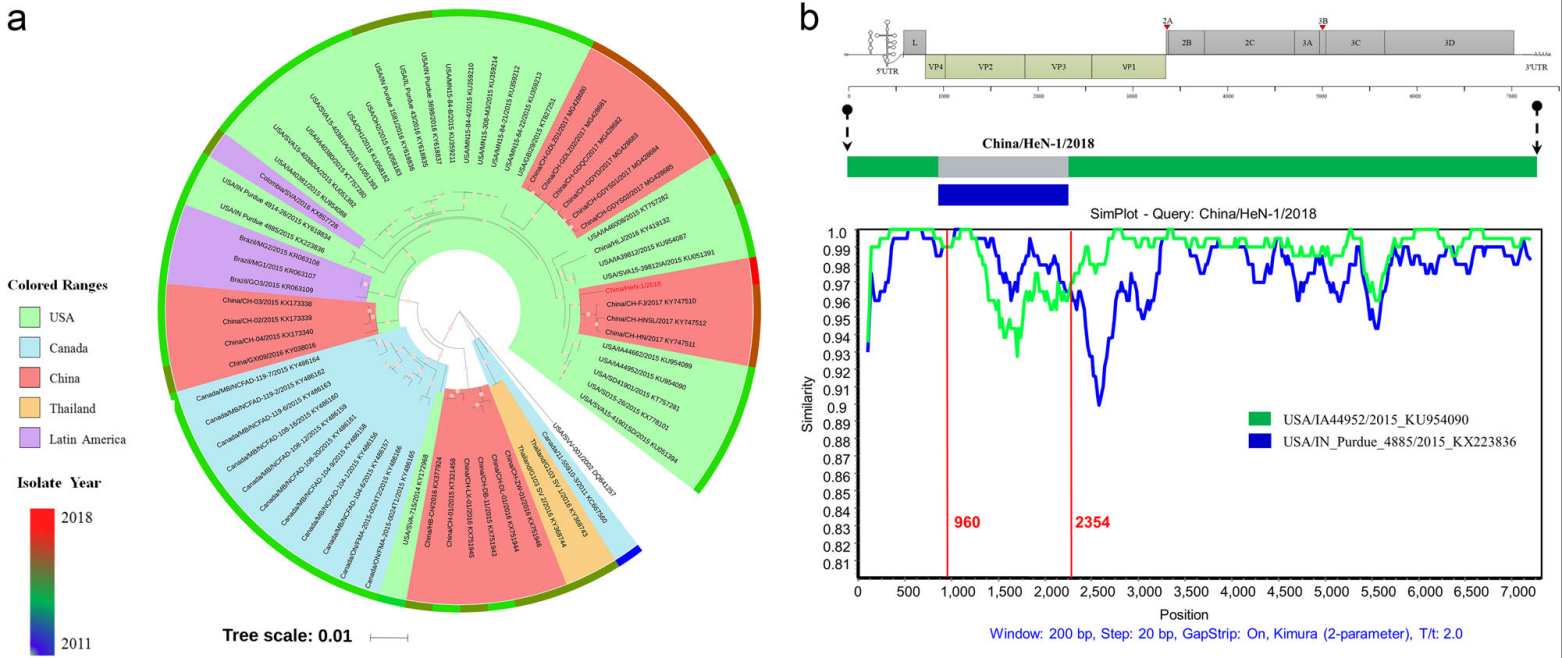
Phylogenetic and recombination analyses of the whole-genome nucleotide sequence of China/HeN-1/2018. **a** Phylogenetic analysis based on the whole genomes of China/HeN-1/2018 and other SVV isolates, available from GenBank. The phylogenetic tree was constructed by the MEGA 6.0 software using the maximum-likelihood method with 1000 bootstrap replicates. **b** Recombination analysis based on the whole genome of China/HeN-1/2018. Reference strains, USA/IA44952/2015 (green) and USA/IN_Purdue_4885/2015 (blue), were used as putative parental strains. The *X*-axis indicates the location of the query sequence, and the *Y*-axis indicates the percentage of identity

The complete genome sequence of HeN-1/2018 was determined by overlapping PCR and rapid amplification of cDNA ends (RACE), as previously described^10^. The genome of HeN-1/2018 was found to consist of 7282 nt in length, with the 5′-UTR of 668 nt and 3′-UTR of 68 nt, excluding the poly(A) tail. The genome constitution represented a standard L-4-3-4 picornavirus arrangement, which was similar to that of previously reported SVV strains^3^. Sequence alignment of HeN-1/2018 with all SVV sequences available in GenBank revealed that HeN-1/2018 shared the highest identity (98.4%) with CH-FJ/2017 (isolated in Fujian province in 2017) and 98.3% identity with CH-HN/2017 and CH-HNSL/2017 (both isolated in Henan province in 2017)^11^. Phylogenetic analysis showed that HeN-1/2018 was clustered, together with the above three strains, into the USA SVV branch (Fig. 1a). Interestingly, other SVV strains, isolated from Guangdong, Hunan, and Hubei provinces, were clustered into the Canada SVV branch (Fig. 1a), although these strains were tightly close to HeN-1/2018 geographically and temporally. These results revealed that the SVV strains circulating in China are genetically diverse, which provides a potential platform for recombination.

Given the potential importance of genetic recombination during evolutionary adaptation of picornaviruses^9^, the recombination events among global SVV strains were further investigated using the RDP4 software^12^. Seven different algorithms, including RDP, Bootscan, MaxChi, GENECONV, Chimaera, SiScanand, and 3Seq were employed. The results provided strong statistical support for a HeN-1/2018 recombination event (*P* < 0.01, recombinant score > 0.6) and indicated that this novel strain derived from USA/IA44952/2015 and USA/IN_Purdue_4885/2015, which were isolated in the United States in 2015. Meanwhile, similar genetic recombination patterns among the HeN-1/2018, USA/IA44952/2015, and USA/IN_Purdue_4885/2015 strains were further confirmed by SimPlot and Bootscan analysis. The SimPlot graph clearly revealed the breakpoints that separated the genome of HeN-1/2018 into three regions, of which two fragments arose from USA/IA44952/2015 (regions 1–959 nt and 2355–7312 nt) and one fragment originated from USA/IN_Purdue_4885/2015 (region 960–2354 nt) (Fig. 1b). Consistent with the above recombination analysis data, in the past decade, China has imported the largest number of breeding pigs from the United States, accounting for 38.29% of total imports (data from the Annual Report on China’s Swine Industry, 2017). Based on the organization of the HeN-1/2018 genome, the shortest recombinant fragment included the region of the VP4 (partial), VP2, and VP3 (partial) genes. All of the above data implied that genetic recombination of SVV had occurred in China.

Genetic recombination is generally considered to be the major mechanism of the evolution of picornaviruses and has been widely regarded as a common phenomenon among viruses in this family, such as FMDV, enterovirus 71, and poliovirus^9^. However, there has been no report about recombination of SVV until now. Herein, we demonstrated, for the first time, genetic recombination of SVV and the diversity of SVV strains circulating in China. Considering the rapid development of the pig industry in China, frequent movements of live pigs through different regions and importation of live pigs from foreign countries, different SVVs are easily introduced into pig populations at pig farms with a low biosecurity level, potentially causing continuous emergence of SVV variants in China. Therefore, urgent surveillance on incidences of SVV infection and proper preventive measures should be carried out in China to limit a large-scale spread of SVV in the future.
